# Racial Disparities in Post-Acute Home Health Care Referral and Utilization among Older Adults with Diabetes

**DOI:** 10.3390/ijerph18063196

**Published:** 2021-03-19

**Authors:** Jamie M. Smith, Olga F. Jarrín, Haiqun Lin, Jennifer Tsui, Tina Dharamdasani, Charlotte Thomas-Hawkins

**Affiliations:** 1 College of Nursing, Thomas Jefferson University, Philadelphia, PA 19107, USA; jamie.smith3@jefferson.edu; 2 School of Nursing, Rutgers, The State University of New Jersey, Newark, NJ 07108, USA; haiqun.lin@rutgers.edu (H.L.); charlot@sn.rutgers.edu (C.T.-H.); 3 Institute for Health, Health Care Policy, and Aging Research, Rutgers, The State University of New Jersey, New Brunswick, NJ 08901, USA; 4 School of Public Health, Rutgers, The State University of New Jersey, Piscataway, NJ 08854, USA; tina.dharamdasani@rutgers.edu; 5 Keck School of Medicine of USC, University of Southern California, Los Angeles, LA 90033, USA; tsuijenn@usc.edu

**Keywords:** chronic conditions, diabetes, older adults, race or ethnicity, health care access, home health care, social determinants of health, inequalities or inequities

## Abstract

Racial and ethnic disparities exist in diabetes prevalence, health services utilization, and outcomes including disabling and life-threatening complications among patients with diabetes. Home health care may especially benefit older adults with diabetes through individualized education, advocacy, care coordination, and psychosocial support for patients and their caregivers. The purpose of this study was to examine the association between race/ethnicity and hospital discharge to home health care and subsequent utilization of home health care among a cohort of adults (age 50 and older) who experienced a diabetes-related hospitalization. The study was limited to patients who were continuously enrolled in Medicare for at least 12 months and in the United States. The cohort (*n* = 786,758) was followed for 14 days after their diabetes-related index hospitalization, using linked Medicare administrative, claims, and assessment data (2014–2016). Multivariate logistic regression models included patient demographics, comorbidities, hospital length of stay, geographic region, neighborhood deprivation, and rural/urban setting. In fully adjusted models, hospital discharge to home health care was significantly less likely among Hispanic (OR 0.8, 95% CI 0.8–0.8) and American Indian (OR 0.8, CI 0.8–0.8) patients compared to White patients. Among those discharged to home health care, all non-white racial/ethnic minority patients were less likely to receive services within 14-days. Future efforts to reduce racial/ethnic disparities in post-acute care outcomes among patients with a diabetes-related hospitalization should include policies and practice guidelines that address structural racism and systemic barriers to accessing home health care services.

## 1. Introduction

In the United States, an estimated 28% of older adults live with diabetes, a chronic condition contributing to macro- and microvascular complications [1]. Diabetes is associated with more frequent use of emergency and acute care, higher mortality [2,3], longer hospitalizations, and more frequent rehospitalizations [4,5]. For older adults with multiple chronic conditions, post-acute care transitions are a particularly high-risk period for adverse events and rehospitalization [6,7]. Home health care visits are one important aid to effective transition from hospital to home and may reduce adverse events among high-risk patients [8,9,10]. For patients with diabetes, home health visits can be used to individualize teaching and support of patients with management of medications and dietary guidelines to optimize glycemic control and prevent complications [11,12]. The American Diabetes Association recommends health care providers evaluate patients’ diabetes self-management skills following a transition between care settings [13]. Post-acute home health care visits provide opportunities to assess the presence of complicating factors and engage patients and their caregivers in individualized education and support with blood glucose monitoring, insulin administration, nutritional support, diabetic foot care, wound care, and management of other chronic conditions.

Black, Hispanic, Asian American, and Native American adults are more likely to report experiencing discrimination in health care and avoiding health care out of concern for discrimination or poor treatment compared to white adults [14,15,16,17]. Patients’ acceptance of home health care is associated with the quality of hospital discharge planning communication and prior positive or negative experiences with post-acute care [18,19]. Efforts to improve hospital discharge decision-making through clinical decision support may help to better align referral/receipt of services [8,20]. However, any efforts must also consider and address systemic barriers that contribute to ongoing racial/ethnic disparities in current home health care referrals and utilization patterns. Residence in socioeconomically disadvantaged neighborhoods often poses barriers to health-promoting resources and services [21,22]. Characterized by area-level poverty, high unemployment, and decreased educational attainment [23], neighborhood disadvantage is associated with increased rehospitalizations [23,24,25] and poor glucose control in older adults with diabetes [26]. However, the inverse may be true in the case of home health care, due to its inclusion as a core benefit under Medicaid’s community-based long-term services and supports (LTSS).

Differences in insurance coverage for Medicare beneficiaries may impact hospital discharge planning and referral and utilization of home health care services. In the United States, Medicare is the national hospital and medical insurance for older adults and people with long-standing disability and may reimburse for services under a fee-for-service or managed care plan. Medicaid is the medical insurance for people with very low income or disabilities; jointly funded by the federal and state governments. Both Medicaid and fee-for-service (FFS) Medicare programs cover intermittent skilled home health care as a core benefit with no co-pay, deductible, or limits on the service utilization. Until 2020, Medicare required patients to be homebound (unable to safely leave their home on a regular basis without assistance) to receive home health services. In contrast, Medicaid may cover skilled home health care for patients who are able to leave their home but have unreliable transportation or caregiver responsibilities limiting their ability to attend frequent primary care appointments for disease monitoring and management. Medicare Advantage (managed care) differs from FFS Medicare in that the private plans may have a co-pay for home health care and may have a restricted network of home health agency providers, in addition to reimbursing home health agencies for services at a lower rate relative to FFS Medicare [27]. Many older adults can choose between fee-for-service Medicare or a private Medicare Advantage plan, however, dual-eligible Medicare/Medicaid beneficiaries in some states are enrolled exclusively in Medicare Advantage. This study includes patients enrolled in both FFS Medicare and Medicare Advantage, including patients who are dual-eligible for Medicaid.

There are multiple ways for a patient to gain access to post-acute home health care. Discharge planning should be a collaborative function between patient/support system, in-patient providers, and outpatient services. A referral (or discharge disposition) to home health care indicates the discharge plan upon leaving the hospital was home with home health care services. We further examined whether patients received an initial post-acute visit. In coordinated care, we expect congruency between referral and utilization, allowing for a hand off between providers, ability of home health agency to prioritize visits as needed, and limited disruption in patient care. Differences in referral and utilization may indicate missed opportunities for patients to receive expected or needed services. To improve post-acute care outcomes among Medicare beneficiaries with diabetes, it is important to first identify the gaps or inequities in access to care. The purpose of this study was to explore racial/ethnic differences in post-acute home health care referral and utilization following diabetes-related hospitalizations among a cohort of Medicare beneficiaries. We also examined the influences of individual and societal factors on referral and utilization outcomes.

## 2. Materials and Methods

### 2.1. Study Design and Data Sources

We performed a retrospective analysis of Medicare fee-for-service and Medicare Advantage beneficiaries with an index hospital stay related to diabetes in 2015. The primary datasets used in this study were complete for both Medicare fee-for-service (FFS) and Medicare Advantage population. The study design and variable selection were informed by Andersen and Newman’s Framework for Viewing Health Services Utilization [28]. From this lens, societal determinants including federal and state policy regulating health insurance (i.e., Medicare and Medicaid), neighborhood socio-economic factors, and structural racism. The health services system includes care access and quality, availability of culturally and linguistically appropriate services, and systemic and institutional racism. Within this framework, individual determinants—such as Black race or limited-English proficiency—reflect the individual and joint effects of societal determinants (e.g., structural racism) and health services system factors (e.g., institutional racism and access to culturally and linguistically appropriate care).

### 2.2. Study Population

**Figure 1:**
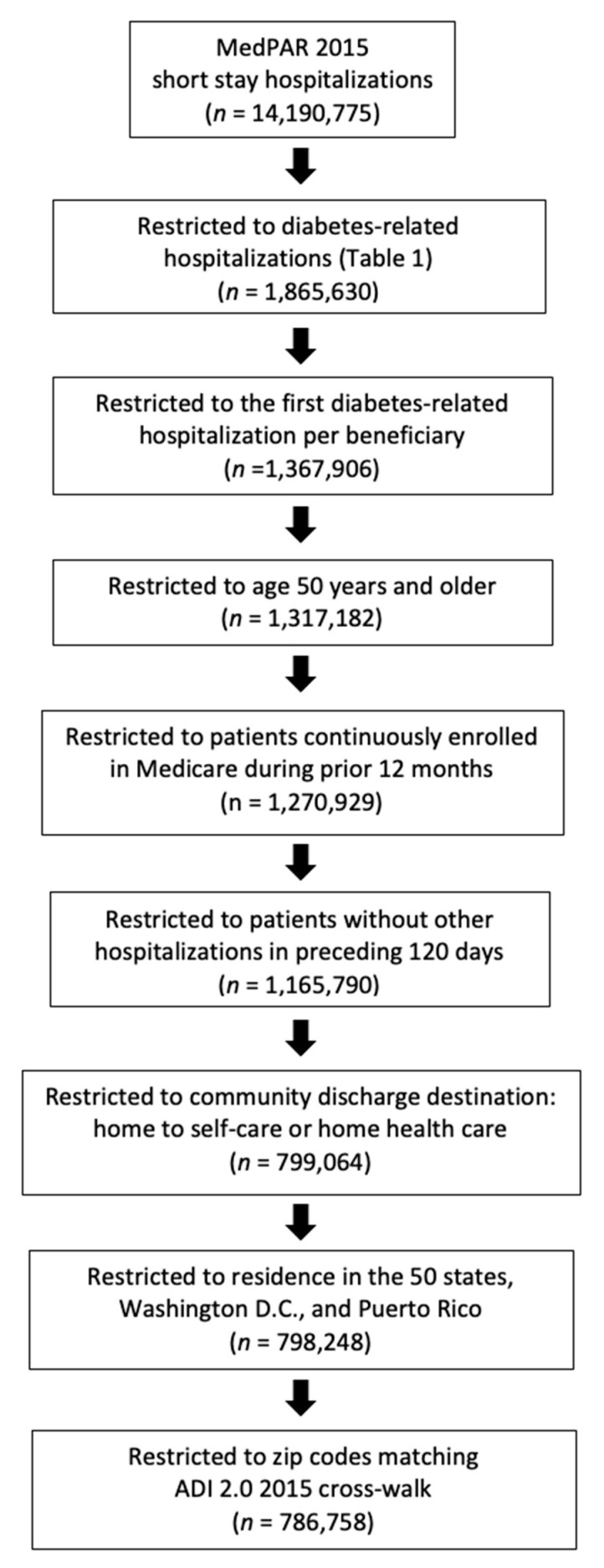
Delimitation of index hospitalizations and study sample.

To create the study sample (Figure 1), we identified all Medicare beneficiaries aged 50 or older who experienced a diabetes-related hospital admission in 2015 (*n* = 1,270,929). Diabetes-related hospitalizations were identified by a primary admitting diagnosis of diabetes or a secondary diagnosis of diabetes combined with a diabetes-related comorbidity [29]. Table 1 displays the list of International Classification of Diseases ICD-9 and ICD-10 diagnoses used to identify diabetes-associated hospitalizations. Index hospitalizations were restricted to patients discharged to home health care or home with self-care, who were continuously enrolled in Medicare for at least 12 months (Figure 1). To minimize the possibility of the index stay itself being a rehospitalization, we additionally excluded patients hospitalized during the 120-days prior to the index hospitalization. Hospital discharge, home health care admission, and rehospitalization outcomes (reported in a separate paper) extended into 2016. Patients aged 50 and older were included in the study sample. Chronic stress, complex health conditions, and social disparities increase patients’ risk for premature age-related morbidities, organ damage, and permanent disability [30].

### 2.3. Outcomes

To better understand the factors impacting utilization of home health care we first evaluated which patients were discharged from the hospital to home health care versus to home with self-care. The main or final outcome was any home health care usage within 14-days of hospital discharge. The Medicare Provider and Analysis Review (MedPAR) file was used to identify hospitalizations, diagnoses, and hospital discharge disposition. Home health care utilization dates relative to the hospitalization (14 days after) was calculated from the home health care Outcome and Assessment Information Set (OASIS) assessments (2015–2016).

**Figure 2:**
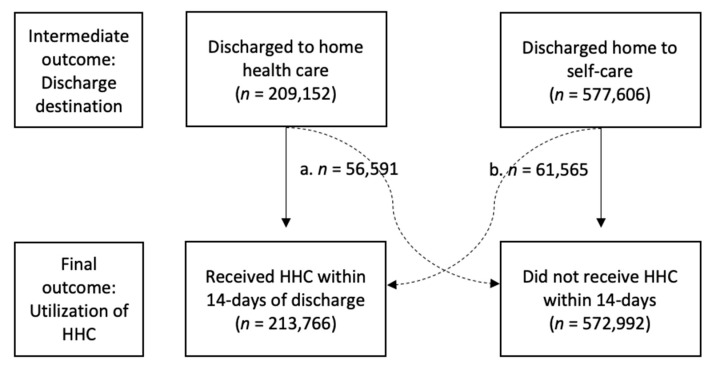
Discharge destination and 14-day home health care (HHC) utilization outcomes for sample (*n* = 786,758). Note: Solid lines represent expected patterns; dashed lines represent unexpected patterns. (**a**) 56,152 (27%) patients who were discharged to HHC did not receive services. (**b**) 61,565 (11%) patients who were discharged home without HHC received services.

Figure 2 shows 209,152 patients (27%) were discharged from the hospital to home health care and 213,766 (27%) received home health care services within 14-days. However, these are not necessarily the same patients, as among those discharged to home health care only 73% received services within 14-days. Additionally, among patients discharged home to self-care 11% received home health care within 14-days.

### 2.4. Individual-Level Variables

The Elixhauser Comorbidity Index Score with readmission weights calculated from index hospital admission diagnosis codes contained in MedPAR was used to calculate clinical complexity [31]. We supplemented the list of Elixhauser comorbidities used in descriptive and multivariate analyses with two additional dummy variables for end-stage renal disease and Alzheimer’s disease and related dementias utilizing chronic condition flags from the Medicare Beneficiary Summary File (MBSF). Demographic data including age, sex, insurance type, and Medicare enrollment status were also obtained from the MBSF. Home health care use during the 120 days prior to index hospitalization was calculated from OASIS assessments (2014–2015). Self-reported race/ethnicity data from OASIS assessments (2013–2016) augmented the imputed Research Triangle Institute (RTI) race variable in the MBSF, minimizing misclassification errors and instances of other/unknown race [32].

### 2.5. Geographic-Level Variables

Neighborhood socioeconomic advantage, rural–urban designation, and region of the country were examined as part of societal determinants of home health care referral and use [24,33]. Socioeconomic advantage was measured with the 2015 Area Deprivation Index 2.0 (ADI 2.0) [34], a composite index of 17 socioeconomic indicators from the 2011–2015 U.S. Census American Community Survey, linked to patients’ nine-digit zip code [24]. A binary variable was created to classify disadvantaged neighborhoods using the 85th percentile (national ranking) of the ADI 2.0 [24]. We then split the neighborhood variable into four categories to distinguish between disadvantaged neighborhoods located in urban or rural areas. The Rural–Urban Continuum Codes (RUCC) codes 1–3 identify urban areas and codes 4–9 areas considered to be rural [35]. A four-category RUCC-ADI combination variable was then created: (a) rural-advantaged, (b) rural-disadvantaged, (c) urban-advantaged, (d) urban-disadvantaged. Finally, we created dummy codes for the nine U.S. Census Division regions and Puerto Rico, linked to patients’ state of residence at the end of 2015.

### 2.6. Analytic Approach

First, we explored patients’ clinical and socio-demographic characteristics, by hospital discharge destination (home health care vs. home to self-care), and by subsequent utilization of home health care. Descriptive results were then stratified by race/ethnicity, neighborhood profiles (rural-advantaged, rural-disadvantaged, urban-advantaged, and urban-disadvantaged), and insurance (Medicare fee-for-service or Medicare Advantage alone or in combination with Medicaid).

We used multivariable logistic regression models to evaluate the predictors of the intermediate outcome: hospital discharge to home health care. The model was controlled for age, sex, race/ethnicity, insurance, prior HHC use, neighborhood profile, and census region). Then to evaluate the final outcome, we used logistic regression models to assess the predictors of utilization of home health care during the initial 14-day post-acute hospital period, stratified by discharge disposition. These models were controlled for age, sex, race/ethnicity, insurance, prior HHC use, neighborhood profile, and census region (Table 3, Models 2a and 2b). The area under the receiver operator curve (c-statistic) was used to estimate model performance. All analyses were performed using SAS software, version (9.4) (SAS Institute Inc., Cary, NC, USA).

## 3. Results

### 3.1. Study Population

We identified 786,758 Medicare beneficiaries with a diagnosis of diabetes who experienced a diabetes-related hospitalization and were discharged to home or home health care services in 2015. The median age of the sample was 73.1 years (IQR = 67–80). The racial/ethnic composition of the sample was two-thirds (68%) (non-Hispanic) White, 17% Black, 11% Hispanic, 2.5% Asian American/Pacific Islander (AAPI), and 1% American Indian/Alaska Native (AIAN). Greater than one-third (36%) of the sample was enrolled in a Medicare Advantage plan, and 30% were dual-eligible for Medicaid. All patients had a diagnosis of diabetes, and 29% had diabetes with chronic complications. Other common comorbidities included hypertension (90%), congestive heart failure (37%), renal failure (37%), fluid and electrolyte disorders (35%), and chronic pulmonary disease (26%). During the 120 days prior to the index hospitalization, 15% of the sample had used home health care. Additional descriptive results are presented in Table S1.

### 3.2. Sample Characteristics Stratified by Race, Neighborhood Profile, and Insurance

Table 2 presents the descriptive results stratified by race and ethnicity. One of the most striking differences is the age distribution by race, with 35% of Black, 33% of AIAN, and 27% of Hispanic patients in the sample under the age of 66, compared to 19% of AAPI, and 18% of white beneficiaries. Residence in a socioeconomically disadvantaged zip code (ADI 2.0 national ranking at or above 85th percentile) was more prevalent among Black (32%), AIAN (29%), and Hispanic (26%) patients, compared to White (12%) and AAPI (7%) patients. Slightly more than one-third (36%) of the sample was enrolled in a Medicare Advantage plan, with greater enrollment among Hispanic patients (52%), and lower enrollment among AIAN patients (17%). Medicaid eligibility also differed by race/ethnicity, ranging from 54% of both Hispanic and AAPI patients, to 47% of Black patients, 43% of AIAN patients, and 20% of White patients. Enrollment in Medicare Advantage was less common in rural (24%) compared to urban areas, where enrollment reached 46% in disadvantaged neighborhoods. See Supplemental Table S2 for additional detail on sample characteristics stratified by neighborhood profiles and Supplemental Table S3 for additional detail on sample characteristics stratified by insurance.

### 3.3. Predictors of Hospital Discharge to Home Health Care

Table 3 displays the unadjusted (bivariate) and adjusted logistic regression models (Model 1) estimated to assess the influence of study variables on discharge destination to home with a referral for home health care services. In bivariate models, relative to white patients (reference group), hospital discharge/referral to home health care was similar for AAPI patients (OR 1.0, 95% CI 1.0–1.0), greater for Black patients (OR 1.1, 95% CI 1.1–1.1), and lower for Hispanic (OR 0.8, 95% CI 0.8–0.8) and AIAN patients (OR 0.7, 95% CI 0.7–0.7). Additional bivariate models show more frequent hospital discharge/referral to home health care among patients with prior home health care use (OR 3.7, 95% CI 3.7–3.8), age greater than 86 years (OR 2.4, 95% CI 2.4–2.5), dual-eligible (OR 1.3, 95% CI 1.3–1.4), diagnosis of dementia (OR 2.0, 95% CI 1.9–2.0), and residence in urban or rural, socioeconomically disadvantaged zip codes (OR 1.2, 95% CI 1.2–1.2).

In model 1 (Table 3), we controlled for age, sex, insurance type, race/ethnicity, presence of comorbidities, length of stay, recent use of home health care, neighborhood socioeconomic advantage, rural–urban designation, and geographic region. In the adjusted model, Black patients had similar odds of hospital discharge/referral to home health care (OR = 0.98, 95% CI 0.97–1.00) relative to white patients (reference group), while discharge to home health care was less likely for AAPI (OR 0.91, 95% CI 0.88–0.95), Hispanic (OR 0.81, 95% CI 0.79–0.83), and AIAN patients (OR 0.79, 95% CI 0.74–0.85). Use of home health services during the 120 days prior to the index hospitalization was the strongest predictor of receiving a referral for home health care (OR 3.12, 95% CI 3.08–3.17). Discharge to home health care was less likely for men compared to women (OR 0.86, 95% CI 0.85–0.87). Patients aged 86 and older were more than two and a half times more likely to receive a referral (OR 2.61, 95% CI 2.55–2.67) than patients aged 50 to 65 years old. Patients enrolled in Medicare Advantage, Medicare Advantage/Medicaid, or fee-for-service/Medicaid had greater odds of hospital discharge to home health care than patients with Medicare fee-for-service alone. Patients with a residential nine-digit zip code (neighborhood) classified as socioeconomically disadvantaged were more likely to have a hospital discharge/referral to home health care in both urban (OR 1.13, 95% CI 1.10–1.15) and rural areas (OR 1.11, 95% CI 1.09–1.13) relative to their counterparts living in socioeconomically advantaged neighborhoods. The empirical c-statistic of the multivariable logistic model predicting hospital discharge to home health care was 0.754 (Table 3).

### 3.4. Predictors of Home Health Care Use after Index Hospital Discharge (First 14-Days)

We estimated logistic regression models to examine predictors of home health care utilization for patients discharged to home health care (Table 3, Model 2a), and patients discharged to home with self-care (Model 2b). In bivariate models (not shown), relative to white patients (reference group), home health care utilization was greater for Black patients (OR 1.1, 95% CI 1.1–1.1), and lower for AAPI (OR 0.9, 95% CI 0.9–0.9), Hispanic (OR 0.8, 95% CI 0.8–0.8), and AIAN patients (OR 0.7, 95% CI 0.7–0.7). Both models 2a and 2b were adjusted for age, sex, insurance type, race/ethnicity, presence of comorbidities, length of stay, recent use of home health care, neighborhood socioeconomic advantage, rural–urban designation, and geographic region. The models had modes performance predicting home health utilization among patients discharged to home health care (c-statistic 0.674) and good performance among patients discharged to self-care (c-statistic 0.797) [36].

Among patients discharged from the hospital to home health care (Model 2a), patients with a history of home health care use during the 120 days prior to the index hospitalization were two and half times more likely to receive home health care within 14 days of hospital discharge compared to patients without recent home health care use. Racial/ethnic minority patient groups were less likely to receive home health care services compared to White patients, with the greatest disparity for Hispanic patients (OR 0.66, 95% CI 0.64–0.69). Among the patients discharged to home health care, enrollment in Medicare Advantage was associated with lower home health care use compared to Medicare fee-for-service, including patients dual-eligible for Medicaid (OR 0.48, 95% CI 0.47–0.51) as well as those with Medicare Advantage alone (OR 0.52, 95% CI 0.50–0.53).

Among patients discharged from the hospital to home with self-care (Table 3, Model 2b), a history of home health care use during the four months prior to the index hospitalization was the strongest predictor of post-acute home health care utilization. These patients were nine times more likely to receive home health care during the first two weeks after hospital discharge compared to patients who had no recent use of home health care services (OR 9.02, 95% CI 8.83–9.21). Advanced age was the next strongest predictor of home health care utilization among patients discharged from the hospital without a home health care referral, followed by Medicaid dual-eligibility, and a diagnosis of Alzheimer’s disease and related dementias. In this group, Black patients were more likely to receive home health care compared to White patients.

## 4. Discussion

This research explores racial/ethnic differences in home health care referral and utilization following diabetes-related hospitalizations while adjusting for societal and individual-level characteristics. We observed racial/ethnic disparities in post-acute home health care referral and service utilization. Additionally, other individual- characteristics, prior use of home health care, advanced age, and insurance type were associated with home health care referrals and service use. The strong association between recent home health care use, referral at hospital discharge, and utilization suggest adequate care coordination for existing home health care patients.

Overall, we found that patients from all ethnoracial minority groups other than Black were less likely to receive a home health care referral at hospital discharge than White patients in models adjusted for individual, neighborhood, and regional variables (Table 3). Among patients discharged to home health care, all ethnoracial minority groups (including Black patients) were less likely to receive services compared to White patients (Table 3). Furthermore, Black patients who were discharged from the hospital to self-care were significantly more likely to receive home health care within two weeks of discharge from the index hospitalization, suggesting inadequate discharge planning and care coordination. To our knowledge, no other studies have examined home health care utilization among American Indian/Alaska Native (AIAN) patients; however, disparities in health services utilization have been reported including forgoing medical care [37], lack of primary providers [38], and high rates of hospital readmissions [39], generally attributed to geographic and socioeconomic barriers [40]. Our findings for AAPI and Hispanic patients are consistent with prior studies of Medicare beneficiaries with Hawaiian [41] or national samples [33,42]. Barriers to timely referral and utilization of post-acute home health care may be a contributing factor to the increased risk of severe diabetes-related complications observed in racial and ethnic minorities [43]. Home health care may reduce risk of adverse outcomes if encouraged, prescribed, and provided in a timely and equitable manner. Further examination of informal care, cultural values, and patient preferences may also help to inform transition practices.

In addition, our findings suggest potential system-level barriers to post-acute home health care exist in this population related to insurance plan coverage details. Patients with Medicare Advantage were more likely to receive home health care referrals at hospital discharge compared to fee-for-service beneficiaries, but then were half as likely to receive home health care services. These findings reinforce prior research showing that Medicare Advantage beneficiaries use fewer home health care services [44,45,46] and plans have lower expenditures for patients with diabetes than traditional Medicare [47]. The difference in referral versus utilization observed in our study adds to the literature. Additional research is needed to identify the causes of these differences, but they may in part be due to cost-containment strategies, including requiring prior authorizations, restricting networks, cost-sharing, and utilization reviews [44,48]. We acknowledge that there is heterogeneity among Medicare Advantage plans [44] as well as the role healthcare providers play in decision making for post-acute services [48]. These findings are relevant as enrollment in Medicare Advantage increases. Today, 36% of Medicare beneficiaries are in a Medicare Advantage plan, up from 24% a decade ago [49].

While this study does not explore the underlying causes of the disparities, we believe institutional and structural racism may have contributed to the study findings and should be the focus of future research. Epidemiologist and physician Dr. Camara Jones describes institutionalized racism as “the structure, policies, practices, and norms resulting in differential access to the goods, services, and opportunities of society by race” ([50], p. 11). One implication of this study is recognizing the need for acute care and home health care organizations to examine existing institutional policies within the lens of racial justice. Are there structural barriers in place that prevent patients from accessing equitable discharge planning and transitional care? Since discharge planning is often subjective in nature, clinical decision support programs [8,18] may assist in identifying patients who could benefit from home health care services. Patients who did not have recent use of home health care should be given additional attention at hospital admission and in discharge planning. Next, patients/families are reliant on healthcare providers to provide information about home health care services available to them. Misunderstanding and misconceptions about services can deter patients from accepting services [18,19]. Discharge-planning meetings should consist of bias-free information in the patient’s preferred language. Institutions must provide adequate language services so patients/families can make well-informed decisions, especially in the case of limited English-proficiency when interpreter services should be utilized for discharge planning [51]. Lastly, we found racial/ethnic minority patients with home health care referrals were significantly less likely to receive services compared to white patients. Prior research suggests structural factors that could contribute to inequities observed, for example, limited home health care staffing in particular communities [52] or fewer agencies accepting Medicare Advantage [27]. We need further research to identify contributing factors in this patient population. This must include engaging patients and caregivers. Then we can implement effective interventions to address inequities in post-acute home health care use following a diabetes-related hospitalization.

Our study had several limitations. Unmeasured health system characteristics may contribute to the differences we observed in home health care referral and utilization [9]. For example, hospitals in urban areas serving a high volume of patients are more likely to provide formal care coordination, while hospitals serving communities with high poverty and uninsured rates were significantly less likely to do so [53]. Inclusion of hospital and agency characteristics could further our understanding of these relationships. Additionally, patients’ functional status, living situation, and caregiver availability are important considerations for discharge planning decisions, which we did not include, as this information is available for only the patients who received home health care. We also did not seek to determine if patients met federal and state requirements for home health care services under Medicare or Medicaid [54,55], and were unable to determine if patients refused to accept home health care services. Lastly, while the index hospital admissions occurred in 2015 the data remain relevant today because policy changes (i.e., PDGM, CARES Act) afterward are unlikely to have impacted the racial/ethnic differences we observed in home health care referral.

## 5. Conclusions

Among older adults with a diabetes-associated hospitalization, persistent racial and ethnic disparities were observed in home health care referral and utilization. Disparities in use of home health care services were compounded for patients enrolled in Medicare Advantage plans. Further work to understand and eliminate systemic barriers contributing to racial/ethnic disparities in home health care referral and use of services is needed. Special emphasis should be placed on improving health care system capacity to provide culturally and linguistically appropriate services, awareness of the role of home health care as part of community-based long-term supports and services provided under Medicaid and Medicare special needs plans, and continued elimination of barriers to home health care usage in Medicare Advantage plans. Lastly, we do not fully know how the COVID-19 pandemic will change the landscape of post-acute care in the long term. In the short term, home health agencies face increased expenses and decreased patient volume [56,57]. These challenges could exacerbate the disparities uncovered in this study and therefore are important to factor into policy and practice decisions made in response to the COVID-19 pandemic.

## Figures and Tables

**Table 1:** International Classification of Diseases (ICD) Codes Used to Identify Diabetes-Related Hospitalizations.

Ninth Revision, Clinical Modification (ICD-9-CM)	Tenth Revision, Clinical Modification (ICD-10-CM)
Cardiovascular diseases: 410–414, 3,9891, 426–428, 7850, 7851, 430–438, 401–405, 4372, 415–417, 429, 440–444, 446–448, 451–453, 458–459, 557, 7859, 7865, 7943, 7962	Cardiovascular diseases: G45, 58; I09–13,15, 20–21, 23–28, 44–45, 47–51, 62–63, 65–67, 70–75, 77–78, 80–82, 87, 69, 95, 97, 99; K55; M30–31; R00, 03, 07, 09, 58, 94
Diabetes mellitus: 250xx	Diabetes mellitus: E11xx
Renal diseases: 580–586, 590, 595, 597, 5,9800, 5,9801, 5990	Renal diseases: N00–05, 08, 10, 11, 16–19, 30, 34–35, 37, 39
Lower extremity diseases and complications: 0201, 0210, 0220, 0311, 03285, 035, 0390, 337, 342–344, 354, 355, 3568, 3569, 3572, 3581, 4402, 4423, 4438, 4439, 44422, 44502, 4510, 4512, 454, 680–682, 684–686, 690, 694–698, 700–703, 707, 709, 711, 7184, 7271, 730, 735, 736, 7396, 7854	Lower extremity diseases and complications: A20, 21, 31, 36; E08–11, 13, 83; G56–60, 73, 80–83, 90; I83, 96; L00–03, 05, 08–13, 20–21, 26, 28–30, 40, 42–44, 49, 51–53, 57, 60, 66, 71, 80–85, 87, 89–95, 97–98; M00–02, 20–21, 24, 46, 57, 66, 83, 85–87, 89–92
Eye diseases and vision defects: 361, 362, 365–369	Eye diseases and vision defects: E113, 09–11, 13; H26, 28, 36, 250–252, 258–262, 311, 330–334, 338, 340–2, 348–359, 400–406, 408–9, 520, 523–527, 530–536, 538–548; Q150
Mycoses: 110–112, 1141, 1143, 1149, 115–118	Mycoses: B35–49
Fluid and electrolytes: 276	Fluid and electrolytes: E86–87

Note: As of 1 October 2015, the United States transitioned to the ICD-10-CM coding system. Diabetes-related hospitalizations were defined by a primary admitting diagnosis of diabetes or a secondary diagnosis of diabetes combined with a diabetes-related condition including cardiovascular, renal, lower extremity, or eye disease [29].

**Table 2:** Sample characteristics by race/ethnicity, column percentage unless otherwise noted.

	White	Black	Hispanic	AAPI	AIAN
	*n* = 534,733	*n* = 134,250	*n* = 86,834	*n* = 19,888	*n* = 5859
Sex, Male %	53.8	41.7	49.1	50.1	48.0
Age ^1^, %					
50–65	17.6	34.9	26.5	18.9	32.8
66–75	39.3	34.5	35.6	34.7	37.1
76–85	31.0	22.7	28.0	31.9	23.8
86+	12.1	8.0	9.9	14.5	6.3
Insurance, %					
Medicare FFS	54.6	32.1	20.2	27.1	47.0
FFS + Medicaid	13.7	27.3	27.6	34.0	35.8
Medicare Advantage (MA)	25.0	20.8	25.5	19.2	10.1
MA + Medicaid	6.7	19.8	26.7	19.7	7.1
RUCC-ADI 2.0, %					
Rural-Advantaged	17.4	6.0	3.6	2.9	26.8
Rural-Disadvantaged	4.0	5.4	2.5	0.4	17.7
Urban-Advantaged	71.1	61.8	70.7	89.9	44.3
Urban-Disadvantaged	7.5	26.7	23.2	6.8	11.2
Elixhauser score ^2^ (mean)	27.4	30.7	27.0	29.7	29.1
Common Comorbidities, %					
Chronic pulmonary	28.4	24.6	18.2	18.2	25.8
Congestive heart	37.8	40.3	31.5	35.8	35.0
Dementia	15.8	18.0	17.5	19.1	15.7
Depression	13.0	7.8	9.1	5.7	11.3
Diabetes complication	27.5	32.6	35.0	34.0	35.7
Fluid/electrolyte	33.6	38.3	34.9	42.6	38.6
Hypertension	88.3	94.3	91.4	92.4	88.0
Hypothyroidism	20.4	10.0	16.2	14.0	18.3
Peripheral vascular	17.7	15.7	17.6	15.4	16.3
Renal failure	34.6	45.9	38.5	45.6	42.4
Prior HHC (120-days), %	14.6	18.7	16.5	12.6	13.2
Hospital LoS ^3^ (mean)	3.8	4.0	4.2	3.9	3.9

Note: AAPI = Asian American/Pacific Islander; AIAN = American Indian/Alaska Native, FFS = fee for service. ^1^ Age (median years, IQR): 74, 68–81; 70, 64–77; 72, 65–79; 74, 68–82; 70, 65–77. ^2^ Elixhauser score (median, IQR): 26, 14–39; 30, 18–42; 25, 13–39; 28, 15–42; 28, 15–41. ^3^ Hospital length of stay (median days, IQR): 3, 2–5; 3, 2–5; 3, 2–5; 3, 2–5; 3, 2–5.

**Table 3:** Logistic models predicting hospital discharge to home health care (HHC) and use of HHC within 14-days.

	Discharge to HHC		HHC Use within 14-Days	
	Unadjusted	Model 1	Model 2a Discharged to Home Health Care	Model 2b Discharged to Self-Care
		c = 0.754	c = 0.674	c = 0.797
	OR, 95% CI	OR, 95% CI	OR, 95% CI	OR, 95% CI
Race (reference group = white)				
Black	1.1, 1.1–1.1 *	1.0, 1.0–1.0 *	0.9, 0.8–0.9 *	1.2, 1.2–1.2 *
Hispanic	0.8, 0.8–0.8 *	0.8, 0.8–0.8 *	0.7, 0.6–0.7 *	1.0, 1.0–1.0
AAPI	1.0, 1.0–1.0	0.9, 0.9–1.0	0.8, 0.8–0.9 *	1.0, 0.9–1.0
AIAN	0.7, 0.6–0.7 *	0.8, 0.7–0.9 *	0.8, 0.7–0.9 *	0.8, 0.7–0.9 *
Sex (reference = female)				
Male	0.8, 0.8–0.8 *	0.9, 0.9–0.9 *	1.0, 0.9–1.0 *	0.8, 0.8–0.9 *
Age (ref = 50–65 years)				
66–75	1.0, 1.0–1.0 *	1.2, 1.2–1.2 *	1.0, 1.0–1.1 *	1.2, 1.1–1.2 *
76–85	1.5, 1.5–1.5 *	1.7, 1.7–1.7 *	1.3, 1.2–1.3 *	1.7, 1.7–1.8 *
86+	2.4, 2.4–2.5 *	2.6, 2.6–2.7 *	1.4, 1.3–1.4 *	2.5, 2.4–2.6 *
Insurance (reference = FFS only)				
Medicare FFS + Medicaid	1.3, 1.3–1.4 *	1.2, 1.2–1.2 *	1.0, 1.0–1.0	1.4, 1.4–1.4 *
Medicare Advantage (MA)	1.0, 1.0–1.1 *	1.2, 1.2–1.2 *	0.5, 0.5–0.5 *	1.1, 1.0–1.1 *
MA + Medicaid	1.3, 1.3–1.3 *	1.3, 1.3–1.3 *	0.5, 0.5–0.5 *	1.1, 1.1–1.2 *
Prior HHC (ref = no)				
HHC in prior 120 days	3.7, 3.7–3.8 *	3.1, 3.1–3.2 *	2.5, 2.4–2.5 *	9.0, 8.8–9.2 *
RUCC-ADI 2.0 (reference = Rural-Advantaged)				
Rural-Disadvantaged	1.2, 1.2–1.2 *	1.1, 1.1–1.1 *	1.1, 1.0–1.1	1.2, 1.1–1.2 *
Urban-Advantaged	1.1, 1.0–1.1 *	1.1, 1.0–1.1 *	0.9, 0.8–0.9 *	1.1, 1.0–1.1 *
Urban-Disadvantaged	1.2, 1.2–1.2 *	1.1, 1.1–1.2 *	0.9, 0.8–0.9 *	1.1, 1.1–1.2 *

Note: FFS = fee for service; * *p* < 0.05. Models 1, 2a and 2b are adjusted for prior home health care utilization, comorbidities, and census region.

## Data Availability

Restrictions apply to the availability of these data. Data were obtained from CMS/ResDAC and are available from the authors with the permission of CMS/ResDAC.

## Supplementary Materials

The following are available online at https://www.mdpi.com/1660-4601/18/6/3196/s1, Table S1: Sample Characteristics Overall, by Discharge Destination, and Use of Home Health Care (HHC) within 14 Days of Index Hospitalization; Table S2: Sample Characteristics Stratified by Neighborhood Profile based on Rural–Urban Designation and the Area Deprivation Index 2.0; Table S3: Sample Characteristics Stratified by Insurance Type.

## References

[1] Centers for Disease Control (2020). National Diabetes Statistics Report. https://www.cdc.gov/diabetes/pdfs/data/statistics/national-diabetes-statistics-report.pdf.

[2] Collins J., Abbass I.M., Harvey R., Suehs B., Uribe C., Bouchard J., Prewitt R., DeLuzio T., Allen E. (2017). Predictors of all-cause 30 day readmission among Medicare patients with type 2 diabetes. Curr. Med. Res. Opin..

[3] O’Neill K.N., McHugh S.M., Tracey M.L., Fitzgerald A.P., Kearney P.M. (2018). Health service utilization and related costs attributable to diabetes. Diabet. Med..

[4] Enomoto L.M., Shrestha D.P., Rosenthal M.B., Hollenbeak C.S., Gabbay R.A. (2017). Risk factors associated with 30-day readmission and length of stay in patients with type 2 diabetes. J. Diabetes Complicat..

[5] Ostling S., Wyckoff J., Ciarkowski S.L., Pai C., Choe H.M., Bahl V., Gianchandani R. (2017). The relationship between diabetes mellitus and 30-day readmission rates. Clin. Diabetes Endocrinol..

[6] LaManna J.B., Bushy A., Norris A.E., Chase S.K. (2016). Early and intermediate hospital-to-home transition outcomes of older adults diagnosed with diabetes. Diabetes Educ..

[7] Lysy Z., Fung K., Giannakeas V., Fischer H.D., Bell C.M., Lipscombe L.L. (2019). The association between insulin initiation and adverse outcomes after hospital discharge in older adults: A population-based cohort study. J. Gen. Intern. Med..

[8] Topaz M., Trifilio M., Maloney D., Bar-Bachar O., Bowles K.H. (2018). Improving patient prioritization during hospital-homecare transition: A pilot study of a clinical decision support tool. Res. Nurs. Health.

[9] Murtaugh C.M., Deb P., Zhu C., Peng T.R., Barrón Y., Shah S., Moore S.M., Bowles K.H., Kalman J., Feldman P.H. (2017). Reducing readmissions among heart failure patients discharged to home health care: Effectiveness of early and intensive nursing services and early physician follow-up. Health Serv. Res..

[10] Wang J., Liebel D.V., Yu F., Caprio T.V., Shang J. (2019). Inverse dose-response relationship between home health care services and rehospitalization in older adults. J. Am. Med. Dir. Assoc..

[11] Hildebrand J.A., Billimek J., Lee J.A., Sorkin D.H., Olshansky E.F., Clancy S.L., Evangelista L.S. (2020). Effect of diabetes self-management education on glycemic control in Latino adults with type 2 diabetes: A systematic review and meta-analysis. Patient Educ. Couns..

[12] Cunningham A.T., Crittendon D.R., White N., Mills G.D., Diaz V., LaNoue M.D. (2018). The effect of diabetes self-management education on HbA1c and quality of life in African-Americans: A systematic review and meta-analysis. BMC Health Serv. Res..

[13] Beck J., Greenwood D.A., Blanton L., Bollinger S.T., Butcher M.K., Condon J.E., Cypress M., Faulkner P., Fischl A.H., Francis T. (2017). 2017 national standards for diabetes self-management education and support. Diabetes Spectr..

[14] Bleich S.N., Findling M.G., Casey L.S., Blendon R.J., Benson J.M., Steelfisher G.K., Sayde J.M., Miller C. (2019). Discrimination in the United States: Experiences of Black Americans. Health Serv. Res..

[15] Findling M.G., Casey L.S., Fryberg S.A., Hafner S., Blendon R.J., Benson J.M., Sayde J.M., Miller C. (2019). Discrimination in the United States: Experiences of Native Americans. Health Serv. Res..

[16] Findling M.G., Bleich S.N., Casey L.S., Blendon R.J., Benson J.M., Sayde J.M., Miller C. (2019). Discrimination in the United States: Experiences of Latinos. Health Serv. Res..

[17] McMurtry C.L., Findling M.G., Casey L.S., Blendon R.J., Benson J.M., Sayde J.M., Miller C. (2019). Discrimination in the United States: Experiences of Asian Americans. Health Serv. Res..

[18] Sefcik J.S., Nock R.H., Flores E.J., Chase J.D., Bradway C., Potashnik S., Bowles K.H. (2016). Patient preferences for information on post-acute care services. Res. Gerontol. Nurs..

[19] Sefcik J.S., Ritter A.Z., Flores E.J., Nock R.H., Chase J.D., Bradway C., Potashnik S., Bowles K.H. (2017). Why older adults may decline offers of post-acute care services: A qualitative descriptive study. Geriatr. Nurs..

[20] Bowles K.H., Ratcliffe S.J., Holmes J.H., Keim S., Potashnik S., Flores E., Humbrecht D., Whitehouse C.R., Naylor M.D. (2019). Using a decision support algorithm for referrals to post-acute care. J. Am. Med. Dir. Assoc..

[21] Gaskin D.J., Roberts E.T., Chan K.S., McCleary R., Buttorff C., Delarmente B.A. (2019). No man is an island: The impact of neighborhood disadvantage on mortality. Int. J. Environ. Res. Public Health.

[22] Tsui J., Hirsch J.A., Bayer F.J., Quinn J.W., Cahill J., Siscovick D., Lovasi G.S. (2020). Patterns in geographic access to health care facilities across neighborhoods in the United States based on data from the National Establishment Time-Series between 2000 and 2014. JAMA Netw. Open.

[23] Kind A.J.H., Golden R.N. (2018). Social determinants of health: Fundamental drivers of health inequity. WMJ.

[24] Kind A.J.H., Jencks S., Brock J., Yu M., Bartels C., Ehlenbach W., Greenberg C., Smith M. (2014). Neighborhood socioeconomic disadvantage and 30-day rehospitalization: A retrospective cohort study. Ann. Intern. Med..

[25] Joynt Maddox K.E., Chen L.M., Zuckerman R., Epstein A.M. (2018). Association between race, neighborhood, and Medicaid enrollment and outcomes in Medicare home health care. J. Am. Geriatr. Soc..

[26] Durfey S.N.M., Kind A.J.H., Buckingham W.R., DuGoff E.H., Trivedi A.N. (2019). Neighborhood disadvantage and chronic disease management. Health Serv. Res..

[27] Medicare Payment Advisory Commission (2020). Report to the Congress: Medicare Payment Policy.

[28] Andersen R., Newman J.F. (1973). Societal and individual determinants of medical care utilization in the United States. Milbank Meml. Fund Q. Health Soc..

[29] Jiang H.J., Andrews R., Stryer D., Friedman B. (2005). Racial/ethnic disparities in potentially preventable readmissions: The case of diabetes. Am. J. Public Health.

[30] Ogunwole S.M., Golden S.H. (2021). Social determinants of health and structural inequities- Root causes of diabetes disparities. Diabetes Care.

[31] Moore B.J., White S., Washington R., Coenen N., Elixhauser A. (2017). Identifying increased risk of readmission and in-hospital mortality using hospital administrative data: The AHRQ Elixhauser Comorbidity Index. Med. Care.

[32] Jarrín O.F., Nyandege A.N., Grafova I.B., Dong X., Lin H. (2020). Validity of race and ethnicity codes in Medicare administrative data compared with gold-standard self-reported race collected during routine home health care visits. Med. Care.

[33] Jones C.D., Wald H.L., Boxer R.S., Masoudi F.A., Burke R.E., Capp R., Coleman E.A., Ginde A.A. (2017). Characteristics associated with home health care referrals at hospital discharge: Results from the 2012 National Inpatient Sample. Health Serv. Res..

[34] University of Wisconsin School of Medicine and Public Health (2020). Neighborhood Atlas. https://www.neighborhoodatlas.medicine.wisc.edu/.

[35] U.S. Department of Agriculture (2013). Rural-Urban Continuum Codes Documentation. https://www.ers.usda.gov/data-products/rural-urban-continuum-codes/documentation/.

[36] Hosmer D.W., Lemeshow S., Sturdivant R.X. (2013). Applied Logistic Regression.

[37] Towne S.D., Bolin J., Ferdinand A., Nicklett E.J., Smith M.L., Ory M.G. (2017). Assessing diabetes and factors associated with foregoing medical care among persons with diabetes: Disparities facing American Indian/Alaska Native, Black, Hispanic, low income, and Southern adults in the U.S. (2011–2015). Int. J. Environ. Res. Public Health.

[38] Adakai M., Sandoval-Rosario M., Xu F., Aseret-Manygoats T., Allison M., Greenlund K.J., Barbour K.E. (2018). Health disparities among American Indians/Alaska Natives—Arizona, 2017. MMWR Morb. Mortal. Wkly. Rep..

[39] King C., Atwood S., Lozada M., Nelson A.K., Brown C., Sabo S., Curley C., Muskett O., Orav E.J., Shin S. (2018). Identifying risk factors for 30-day readmission events among American Indian patients with diabetes in the Four Corners region of the southwest from 2009 to 2016. PLoS ONE..

[40] Franz C., Atwood S., Orav E.J., Curley C., Brown C., Trevisi L., Nelson A.K., Begay M., Shin S. (2020). Community-based outreach associated with increased health utilization among Navajo individuals living with diabetes: A matched cohort study. BMC Health Serv. Res..

[41] Gandhi K., Lim E., Davis J., Chen J.J. (2018). Racial disparities in health service utilization among Medicare fee-for-service beneficiaries adjusting for multiple chronic conditions. J. Aging Health.

[42] Li J., Qi M., Werner R.M. (2020). Assessment of receipt of the first home health care visit after hospital discharge among older adults. JAMA Netw. Open.

[43] Hazel-Fernandez L., Li Y., Nero D., Moretz C., Slabaugh L., Meah Y., Baltz J., Costantino M., Patel N.C., Bouchard J. (2015). Racial/ethnic and gender differences in severity of diabetes-related complications, health care resource use, and costs in a Medicare population. Popul. Health Manag..

[44] Li Q., Rahman M., Gozalo P., Keohane L.M., Gold M.R., Trivedi A.N. (2018). Regional variations: The use of hospitals, home health, and skilled nursing in traditional Medicare and Medicare Advantage. Health Aff..

[45] Skopec L., Huckfeldt P.J., Wissoker D., Aarons J., Dey J., Oliveira I., Zuckerman S. (2020). Home health and postacute care use in Medicare Advantage and traditional Medicare. Health Aff..

[46] Skopec L., Zuckerman S., Aarons J., Wissoker D., Huckfeldt P.J., Feder J., Berenson R.A., Dey J., Oliveira I. (2020). Home health use in Medicare Advantage compared to use in traditional Medicare. Health Aff..

[47] Mahmoudi E., Tarraf W., Maroukis B.L., Levy H.G. (2016). Does Medicare Managed Care reduce racial/ethnic disparities in diabetes preventive care and healthcare expenditures?. Am. J. Manag. Care.

[48] Li Q., Keohane L.M., Thomas K., Lee Y., Trivedi A.N. (2017). Association of cost sharing with use of home health services among Medicare Advantage enrollees. JAMA Intern. Med..

[49] Freed M., Damico A., Neuman T. (2020). A Dozen Facts about Medicare Advantage in 2020. https://www.kff.org/medicare/issue-brief/a-dozen-facts-about-medicare-advantage-in-2020/.

[50] Jones C.P. (2002). Confronting institutionalized racism. Phylon.

[51] Golden S.H., Joseph J.J., Hill-Briggs F. (2021). Casting a health equity lens on endocrinology and diabetes. J. Clin. Endocrinol. Metab..

[52] Davitt J.K., Bourjolly J., Frasso R. (2015). Understanding inequities in home health care outcomes staff views on agency and system factors. Res. Gerontol. Nurs..

[53] Chen J., DuGoff E.H., Novak P., Wang M.Q. (2020). Variation of hospital-based adoption of care coordination services by community-level social determinants of health. Health Care Manag. Rev..

[54] Kaiser State Health Facts (2020). Medicaid & CHIP. https://www.kff.org/state-category/medicaid-chip/.

[55] Center for Medicare and Medicaid Services (2019). Important Message about the Jimmo Settlement. https://www.cms.gov/Center/Special-Topic/Jimmo-Center.

[56] Jones C., Bowles K. (2020). Emerging challenges and opportunities for home health care in the time of COVID-19. J. Am. Med. Dir. Assoc..

[57] Shang J., Chastain A.M., Perera U.G.E., Quigley D.D., Fu C.J., Dick A.W., Pogorzelska-Maziarz M., Stone P.W. (2020). COVID-19 preparedness in US home health care agencies. J. Am. Med. Dir. Assoc..

