# Supplementary material for: Racial Disparities in Post-Acute Home Health Care Referral and Utilization among Older Adults with Diabetes
Source: Int J Environ Res Public Health. 2021 Mar 19;18(6):3196. doi: 10.3390/ijerph18063196 (PMC8003472; doi:10.3390/ijerph18063196)
Supplement: Supplementary file 1 [file ijerph-18-03196-s001.pdf]

Table S1. Sample Characteristics Overall, by Discharge Destination, and Use of Home Health Care (HHC) within 14 Days of Index Hospitalization, column percent unless otherwise noted.

Characteristics	Total	Discharged to HHC	Received HHC
	N = 786,758	*n* = 209,152	*n* = 213,766
Race/Ethnicity, %			
White, non-Hispanic	68.0	69.0	68.7
Black	17.1	18.2	18.6
Hispanic	11.0	9.5	9.7
Asian/Pacific Islander	2.5	2.5	2.3
American Indian/Alaska Native	0.7	0.6	0.6
Unknown/Other	0.7	0.2	---
Sex, %			
Male	51.2	46.3	45.3
Age, %			
50-65	21.7	18.2	18.5
66-75	38.0	32.1	31.0
76-85	29.1	32.6	33.0
86+	11.1	17.2	17.5
Insurance, %			
Fee-for-Service (FFS)	46.2	43.1	45.0
FFS and Medicaid	18.2	20.9	23.4
Medicare Advantage (MA)	24.1	23.0	20.1
MA and Medicaid	11.5	13.0	11.5
RUCC-ADI 2.0 (zip code level), %			
Rural-Advantaged (ADI 2.0 <85)	13.6	12.0	12.6
Rural-Disadvantaged (ADI 2.0 ≥85)	4.1	3.8	4.3
Urban-Advantaged (ADI 2.0 <85)	69.8	71.6	70.0
Urban-Disadvantaged (ADI 2.0 ≥85)	12.5	12.7	13.1
Elixhauser score ($\bar{x}$)	16.5	16.6	16.5
Comorbidities, %			
Chronic pulmonary disease	26.2	29.3	29.7
Congestive heart failure	37.4	44.3	44.6
Dementia	16.4	23.6	25.6
Depression	11.4	12.9	12.9
Diabetes with complications	29.4	34.9	33.9
Fluid and electrolyte disorders	34.8	40.5	39.7
Hypertension	89.8	89.4	89.3
Hypothyroidism	17.9	19.6	20.0
Peripheral vascular disease	17.2	19.4	19.2
Renal failure	37.2	41.7	42.0
Prior HHC use % (120-day look back)	15.4	29.6	36.9
Length of stay, days ($\bar{x}$, *SD*)	3.9 (4.2)	5.2 (4.2)	5.0 (4.1)

Note: Elixhauser score (median, IQR) 26, 14–40; 31, 19–44; 31, 19–43; Length of stay, days (median, IQR) 3, 2–5; 4, 3–6; 4, 3–6; Age (median, IQR) 73, 67–80; 75, 68–83; 76, 68–83.

Table S2. Sample Characteristics Stratified by Neighborhood Profile based on Rural-Urban Designation and the Area Deprivation Index 2.0, row percentage unless otherwise noted

	Rural-Advantaged		Rural-Disadvantaged		Urban-Advantaged		Urban-Disadvantaged	
Total	106,828	13.6%	32,183	4.1%	549,175	69.8%	98,572	12.5%
Race/Ethnicity								
White, non-Hispanic	92,892	17.4%	21,503	4.0%	380,151	71.1%	40,187	7.5%
Black	8,119	6.0%	7,314	5.4%	82,955	61.8%	35,862	26.7%
Hispanic	3,122	3.6%	2,141	2.5%	61,414	70.7%	20,157	23.2%
Asian/Pacific Islander	579	2.9%	71	0.4%	17,886	89.9%	1,352	6.8%
American Indian/Alaska Native	1,568	26.8%	1,039	17.7%	2,598	44.3%	654	11.2%
Sex								
Female	49,349	12.9%	16,575	4.3%	264,023	68.8%	54,020	14.1%
Male	57,479	14.3%	15,608	3.9%	285,152	70.8%	44,552	11.1%
Age								
50–65	22,329	13.1%	9,346	5.5%	109,313	64.0%	29,929	17.5%
66–75	43,049	14.4%	12,322	4.1%	208,527	69.7%	35,351	11.8%
76–85	31,492	13.8%	8,079	3.5%	164,982	72.0%	24,526	10.7%
86+	9,958	11.4%	2,436	2.8%	66,353	75.8%	8,766	10.0%
Insurance, Medicare								
Fee-For-Service (FFS)	59,396	16.3%	13,829	3.8%	261,886	72.0%	28,570	7.9%
FFS and Medicaid (dual)	21,239	14.8%	10,579	7.4%	86,569	60.5%	24,793	17.3%
Medicare Advantage (MA)	20,001	10.6%	4,836	2.6%	141,414	74.7%	23,142	12.2%
MA and Medicaid (dual)	6,192	6.8%	2,939	3.2%	59,306	65.5%	22,067	24.4%
Elixhauser Score ($\bar{x}$, *SD*)	27.1	(16.3)	27.53	(16.1)	27.99	(16.6)	28.82	(16.6)
Comorbidities								
Chronic pulmonary disease	30,142	14.6%	9,514	4.3%	138,819	70.2%	28,011	13.6%
Congestive heart failure	40,804	13.9%	12,379	4.2%	203,896	69.3%	37,029	12.6%
Dementia	16,422	12.8%	5,538	4.3%	90,284	70.2%	16,424	12.8%
Depression	13,053	14.5%	3,542	3.9%	62,386	69.5%	10,849	12.1%
Diabetes with complications	27,397	11.8%	8,568	3.7%	164,250	71.0%	31,095	13.4%
Hypertension	94,434	13.4%	28,649	4.1%	493,443	69.8%	90,053	12.7%
Hypothyroidism	19,816	14.0%	5,404	3.8%	100,627	71.3%	15,263	10.8%

Fluid and electrolyte disorders	35,613	13.0%	11,252	4.1%	191,504	70.0%	35,261	12.9%
Peripheral vascular disease	17,888	13.2%	5,262	3.9%	95,690	70.6%	16,739	12.3%
Renal failure	37,543	12.8%	11,564	3.9%	205,360	70.1%	38,525	13.1%
Discharged to home health care	25,044	12.0%	7,851	3.8%	149,655	71.6%	26,602	12.7%
Prior HHC (120-day look back)	14,787	12.2%	6,054	5.0%	81,949	67.8%	18,034	14.9%
Post-Acute HHC, first 14 days	27,017	12.6%	9,264	4.3%	149,489	69.9%	27,996	13.1%
Hospital Length of Stay ($\bar{x}$, *SD*)	3.7	(3.1)	3.9	(3.3)	3.8	(3.4)	4.1	(3.6)
Region								
Pacific	5,060	5.8%	372	0.4%	76,193	87.8%	5,123	5.9%
East North Central	21,744	16.9%	3,548	11.0%	83,975	65.3%	19,388	15.1%
East South Central	15,417	24.5%	7,998	12.7%	21,775	50.5%	7,728	12.3%
Middle Atlantic	7,900	7.2%	1,121	1.0%	86,293	79.1%	13,753	12.6%
Mountain	4,797	13.2%	1,114	3.1%	26,484	72.9%	3,942	10.9%
New England	3,515	10.9%	193	0.6%	27,299	84.9%	1,142	3.6%
South Atlantic	21,935	12.0%	8,426	4.6%	129,726	70.9%	22,998	23.3%
West North Central	12,523	1.6%	2,435	5.4%	26,509	58.3%	3,988	4.1%
West South Central	13,876	14.8%	6,547	7.0%	57,139	60.8%	16,425	17.5%
Puerto Rico	59	0.7%	429	5.2%	3,740	45.0%	4,075	49.1%

Note: HHC: home health care; AIAN: American Indian/ Alaska Native; Hospital Length of stay (median, IQR) 3, 2–5; 3, 2–5; 3, 2–5; 3, 2–5; Age (median, IQR) 73, 67–79; 71, 65–78; 73, 67–81; 71, 65–78; Elixhauser score (median, IQR) 26, 14–38; 26, 14–39; 26, 14–40; 27, 15–41; Disadvantaged: ADI 2.0 ≥ 85th percentile; Advantaged: ADI 2.0 < 85th percentile

Table S3. Sample Characteristics Stratified by Insurance Type, row percentage unless otherwise noted

	Medicare fee-for-service only		Dual Medicare fee-for-service/Medicaid		Medicare Advantage only		Dual Medicare Advantage/Medicaid	
Total	363,681	46.2%	143,180	18.2%	189,393	24.1%	90,504	11.5%
Race/Ethnicity								
White, non-Hispanic	292,162	54.6%	73,241	13.7%	133,477	25.0%	35,853	6.7%
Black	43,151	32.1%	36,592	27.3%	27,862	20.8%	26,645	19.9%
Hispanic	17,581	20.3%	23,948	27.6%	22,138	25.5%	23,167	26.7%
Asian/Pacific Islander	5,392	27.1%	6,763	34.0%	3,817	19.2%	3,916	19.7%
American Indian/Alaska Native	2,754	47.0%	2,098	35.8%	591	10.1%	416	7.1%
Sex								
Female	157,751	41.1%	87,250	22.7%	83,309	21.7%	55,657	14.5%
Male	205,930	26.2%	55,930	7.1%	106,084	13.5%	34,847	4.4%
Age ($\bar{x}$, *SD*)	74.6	(9.1)	69.5	(10.9)	74.2	(8.7)	70.9	10.2%
50–65	54,820	32.1%	59,089	34.6%	28,892	16.9%	28,116	16.5%
66–75	146,173	48.9%	41,168	13.8%	79,620	26.6%	32,288	10.8%
76–85	115,847	50.6%	30,160	13.2%	60,626	26.5%	22,446	9.8%
86+	46,841	53.5%	12,763	14.6%	20,255	23.2%	7,654	8.8%
RUCC-ADI 2.0								
Rural-Advantaged (ADI 2.0 <85)	59,396	55.6%	21,239	19.9%	20,001	18.7%	6,192	5.8%
Rural-Disadvantaged (ADI 2.0 ≥ 85)	13,829	43.0%	10,579	32.9%	4,836	15.0%	2,939	9.1%
Urban-Advantaged (ADI 2.0 <85)	261,886	47.7%	86,569	15.8%	141,414	25.8%	59,306	10.8%
Urban-Disadvantaged (ADI 2.0 ≥ 85)	28,570	29.0%	24,793	25.2%	23,142	23.5%	22,067	22.4%
Elixhauser score ($\bar{x}$, *SD*)	27.1	(16.4)	30.7	(16.5)	27.1	(16.5)	28.8	(16.5)
Comorbidities								
Chronic pulmonary disease	88,115	42.7%	45,530	22.1%	45,495	22.0%	27,346	13.2%
Congestive heart failure	134,953	45.9%	55,680	18.9%	70,087	23.8%	33,388	11.4%
Dementia	69,500	54.0%	39,160	30.4%	8,642	6.7%	11,366	8.8%
Depression	38,550	42.9%	20,466	22.8%	19,125	21.3%	11,689	13.0%
Diabetes with complications	95,957	41.5%	47,061	20.4%	58,485	25.3%	29,807	12.9%
Fluid and electrolyte disorders	122,590	44.8%	56,854	20.8%	60,701	22.2%	33,485	12.2%
Hypertension	324,962	46.0%	128,659	18.2%	170,711	24.2%	82,247	11.6%
Hypothyroidism	69,082	49.0%	24,142	17.1%	33,517	23.8%	33,517	10.2%

Peripheral vascular disease	60,787	44.8%	23,318	17.2%	36,665	27.0%	14,809	10.9%
Renal failure	132,732	45.3%	58,517	20.0%	68,934	23.5%	32,809	11.2%
Discharged to Home Health Care	90,146	43.1%	43,732	20.9%	48,140	23.0%	27,134	13.0%
Prior HHC (120-day look back)	49,067	40.6%	36,574	30.3%	19,086	15.8%	16,097	13.3%
Post-Acute HHC, first 14 days	96,239	45.0%	50,054	23.4%	42,877	20.1%	24,596	11.5%
Hospital length of stay, days ($\bar{x}$, *SD*)	3.7	(3.2)	3.7	(3.2)	3.9	(3.4)	4.0	(3.6)

Notes: FFS = Fee-for-services; HHC = home health care ; Hospital length of stay, days (median, IQR) 3, 2–5; 3, 2–5; 3, 2–5; 3, 2–5; Age (median, IQR) 74, 68–81; 68, 61–78; 74, 68–80; 71, 64–78; Elixhauser score (median, IQR) 26, 13–39; 30, 18–42; 25, 13–38; 27, 16–40
